# Janus-faced microglia: beneficial and detrimental consequences of microglial phagocytosis

**DOI:** 10.3389/fncel.2013.00006

**Published:** 2013-01-30

**Authors:** Amanda Sierra, Oihane Abiega, Anahita Shahraz, Harald Neumann

**Affiliations:** ^1^Achucarro—Basque Center for NeuroscienceZamudio, Spain; ^2^Department of Neuroscience, University of the Basque Country EHU/UPVLeioa, Spain; ^3^Ikerbasque—Basque Foundation for ScienceBilbao, Spain; ^4^Neural Reconstruction Group, Institute of Reconstructive Neurobiology, University of BonnBonn, Germany

**Keywords:** microglia, phagocytosis, apoptosis, synapses, debris, myelin, amyloid, inflammation

## Abstract

Microglia are the resident brain macrophages and they have been traditionally studied as orchestrators of the brain inflammatory response during infections and disease. In addition, microglia has a more benign, less explored role as the brain professional phagocytes. Phagocytosis is a term coined from the Greek to describe the receptor-mediated engulfment and degradation of dead cells and microbes. In addition, microglia phagocytoses brain-specific cargo, such as axonal and myelin debris in spinal cord injury or multiple sclerosis, amyloid-β deposits in Alzheimer's disease, and supernumerary synapses in postnatal development. Common mechanisms of recognition, engulfment, and degradation of the different types of cargo are assumed, but very little is known about the shared and specific molecules involved in the phagocytosis of each target by microglia. More importantly, the functional consequences of microglial phagocytosis remain largely unexplored. Overall, phagocytosis is considered a beneficial phenomenon, since it eliminates dead cells and induces an anti-inflammatory response. However, phagocytosis can also activate the respiratory burst, which produces toxic reactive oxygen species (ROS). Phagocytosis has been traditionally studied in pathological conditions, leading to the assumption that microglia have to be activated in order to become efficient phagocytes. Recent data, however, has shown that unchallenged microglia phagocytose apoptotic cells during development and in adult neurogenic niches, suggesting an overlooked role in brain remodeling throughout the normal lifespan. The present review will summarize the current state of the literature regarding the role of microglial phagocytosis in maintaining tissue homeostasis in health as in disease.

## Definition of phagocytosis

Phagocytosis is a Greek-derived term which literally means the cellular process of eating. It describes the recognition, engulfment, and degradation of large (>0.5 μm), particulated organisms or structures (Mukherjee et al., 1997). Phagocytosis of invading microorganisms by immune cells was first discovered by the father of cellular immunology Ilya Metchnikoff in 1882, for which he was awarded the Nobel Prize. Most cell types, including unicellular organisms, have the capacity to phagocytose. In multicellular organisms, and more particularly in animals with a well-developed immune system, phagocytosis is mostly performed by specialized, professional phagocytes: macrophages, dendritic cells (DCs), and neutrophils. Together with inflammation, phagocytosis composes the first line of defense against multicellular organisms by the innate immune system. In jawed vertebrates, including mammals, phagocytosis also helps to initiate the more specific adaptive immune response through antigen-presentation to T lymphocytes (Litman et al., 2005). In the mammalian central nervous system (CNS), the innate immune response is orchestrated by microglia.

Microglia are the brain resident macrophages. They are of myeloid origin and share many properties with other well-studied tissue macrophages. However, they have a different origin and are a unique macrophage cell type in the adult organism. Microglia are yolk sac-derived, invade the brain during early embryonic development and then locally proliferate in the brain (Ginhoux et al., 2010; Schulz et al., 2012). In contrast to other yolk sac-derived macrophages, they are not replaced during the postnatal period and later life by liver- or bone marrow-derived macrophages (Hoeffel et al., 2012). Cell biology of phagocytosis has been mainly established on bone marrow-derived tissue macrophages. In this review we directly extrapolate basic phagocytic mechanisms from bone marrow-derived tissue macrophages to the less-known processes of microglia, but would like to point out that these assumed similarities might not fully hold true to the yolk sac-derived microglia. Particularly, yolk sac-derived macrophages including microglia may possibly have different tasks since they are confronted with different target structures, mainly apoptotic cells during developmental tissue remodeling, while bone marrow-derived macrophages have a higher chance to be confronted with pathogens, mainly during defense against invading microbes. Overall, microglial phagocytosis is a relatively unknown process in terms of the receptors involved, the mechanisms of its execution, its beneficial or detrimental consequences, and its ultimate impact in maintaining tissue homeostasis. To further complicate the issue, macrophage phagocytosis has two main targets: dead resident cells (apoptotic or necrotic) and live invading microorganisms; whereas microglial phagocytosis appears to be adapted to the brain environment for remodeling tasks such as engulfment of synapses, axonal and myelin debris, or clearance of proteins with very high turnover such as amyloid beta (Aβ) protein.

One major limitation is that in most studies, microglial phagocytosis is assessed exclusively *in vitro*, utilizing a model of phagocytosis in which primary or transformed microglial cells are “fed” with a variety of targets, ranging from latex beads to primary or transformed apoptotic cells, cell debris, or Aβ. These models have proved to be extremely valuable to study the molecular pathways involved in the recognition, engulfment, and degradation of the targets. However, the lack of molecular tools to specifically block these three steps has precluded our understanding of the global impact of microglial phagocytosis *in vivo*. In fact, many *in vivo* experiments rely on the use of phagocytosis “markers” such as CD68 (ED1 or macrosialin) as a proxy. One major problem is that the function of CD68 is unknown. While located in the lysosomal compartment, anti-CD68 antibodies do not block macrophage phagocytosis *in vitro* (Damoiseaux et al., 1994), and macrophages from mice deficient in CD68 have no defects in phagocytosis of bacteria (Song et al., 2011). In the adult hippocampus, the expression of ED1 in microglia phagocytosing apoptotic cells is similar to non-phagocytic microglia and much lower than the expression induced by inflammatory challenge (Sierra et al., 2010). However, a higher expression of ED1 seems to correlate with the capacity of postnatal microglia to engulf synapses (Schafer et al., 2012). Furthermore, few studies have attempted to quantify the extent of microglial phagocytosis *in vivo* (Ashwell, 1990; Dalmau et al., 2003; Parnaik et al., 2000; Sierra et al., 2010) and many rely on a qualitative observation of microglial engulfment to determine whether microglia is phagocytosing or not.

The rapid clearance time of dead cells is likely behind this qualitative rather than quantitative assessment. *In vitro*, live imaging has determined that the clearance time to completely eliminate an apoptotic cell by microglia is 25–95 min in a co-culture system (Parnaik et al., 2000); *in vivo*, the microglial clearance time of apoptotic cells in physiological conditions has been estimated to be 70–90 min (Sierra et al., 2010). These numbers are in agreement with a general 1–2 h clearance time for macrophages (Henson and Hume, 2006). Thus, the number of apoptotic cells observed in particular time point represents only a small fraction of the actual number of apoptotic cells generated (or total number of cells which disappear). For example, if 400 cells die in a 24 h period, a clearance time of 1.5 h would mean that only 25 dead cells can be observed at any time point [for more details on estimation of the clearance time, see Barres et al. (1992); Sierra et al. (2010)]. The phagocytosis of smaller particles (synapses, axons, protein aggregates) is likely to be even faster. The promptness of microglia to eliminate cell debris is therefore an important parameter to take into account when analyzing the dynamics of cell death and phagocytosis.

In addition to the clearance time, two main parameters to quantify microglial phagocytosis of apoptotic cells *in vivo* using immunofluorescence and confocal microscopy have been established: the phagocytic index, or proportion of apoptotic cells which are three-dimensionally engulfed by microglia over the total number of apoptotic cells; and the phagocytic capacity, or proportion of phagocytosing microglia multiplied by the number of phagocytic pouches (i.e., number of apoptotic cells engulfed) over the total number of microglia (Sierra et al., 2010). More recently, a similar parameter was described to quantify microglial phagocytosis of synaptic inputs (allegedly, presynaptic terminals) using high resolution confocal microscopy and three-dimensional rendering to estimate the volume of internalized inputs over the total volume of microglia (Schafer et al., 2012). Similarly, microglial phagocytosis of axonal or myelin debris can be quantified using confocal microscopy to determine the percentage of microglia containing neurofilament-positive axonal material (Hosmane et al., 2012) or myelin basic protein (MBP) (Nielsen et al., 2009). The utilization of these or similar parameters is a necessary starting point to obtain a quantitative picture of microglial phagocytosis across a range of physiological and pathological conditions, which will help us to address many of the open questions. Over the next sections, we will provide an overall description of the mechanical process of phagocytosis; its beneficial and detrimental consequences; and the particular details of phagocytosis of different targets: cells and cell debris, microorganisms, tumor cells, spines, and Aβ deposits.

## The mechanics of phagocytosis

**Figure 1:**
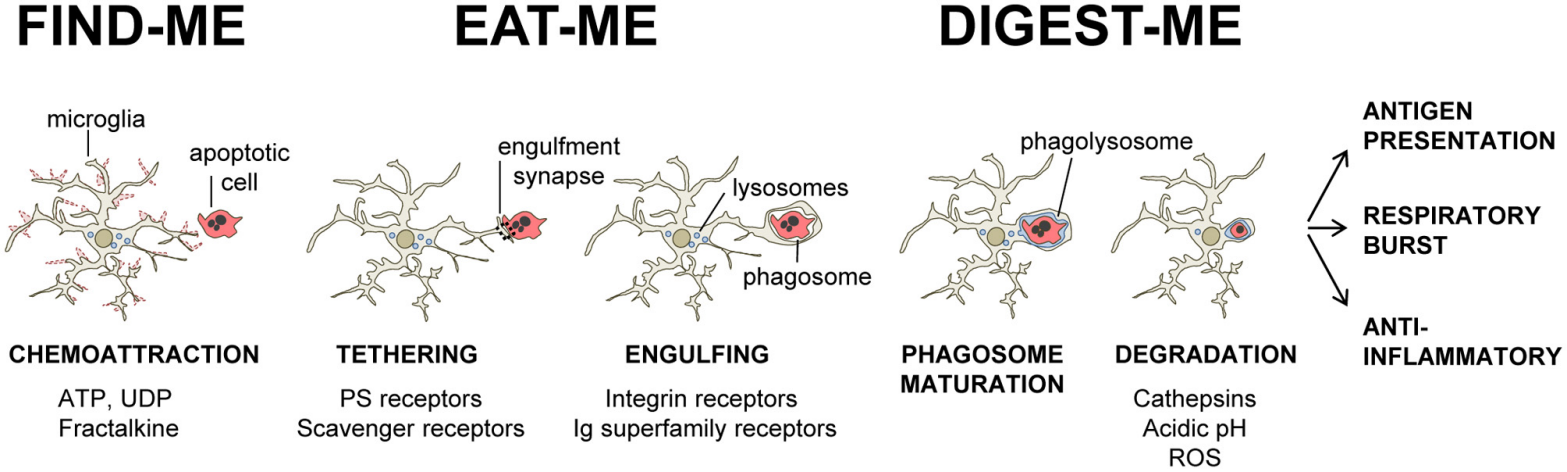
**Three-step model of microglial phagocytosis.** In physiological conditions, microglial processes are highly motile and respond to chemoattractant molecules released by damaged or apoptotic cells (“find-me” signals) such as fractalkine and extracellular nucleotides (ATP, UDP). Next, an engulfment synapse is formed between a series of microglial receptors and their ligands in the membrane of the apoptotic cell (“eat-me” signals), leading to the tethering and engulfing of the apoptotic cell in a phagosome. The phagosome becomes mature by fusing with lysosomes and other organelles, and the apoptotic cell is fully degraded in the phagolysosome in less than 2 h (see text for details).

Our current understanding of the mechanical process of phagocytosis is summarized in the three-step model: find-me, eat-me, and digest-me (Savill et al., 2002), which addresses the engulfment and degradation of apoptotic cells and microorganisms by macrophages and DCs. While it is likely that microglia utilizes similar mechanisms, direct evidence of the molecular details of these events in microglia, beyond the receptors involved in the recognition of the targets, is largely missing. In this section, we will address the featured molecular mechanisms in the process of phagocytosis by microglia (Figure 1).

### Find-me

The process is initiated when the phagocyte encounters a target, either randomly or triggered by signals from the target. Microglial processes are highly motile (Davalos et al., 2005; Nimmerjahn et al., 2005) and their constant surveillance of the brain parenchyma supports their capacity to fortuitously encounter targets to engulf. In addition, apoptotic cells can release signals to attract macrophages and DCs, such as the extracellular nucleotides ATP and UTP (Elliott et al., 2009). In microglia, UDP, the product of degradation of UTP by extracellular ectonucleotidases, acts on P2Y_6_ receptors to facilitate phagocytosis (Koizumi et al., 2007). Another important chemotactic signal released by apoptotic cells is fractalkine/CX3CL1 (Truman et al., 2008; Noda et al., 2011). Microglia express the fractalkine receptor (CX3CR1), which promotes phagocytosis of apoptotic cells (Noda et al., 2011) but shifts microglia toward a phenotype with less capacity to phagocytose Aβ (Lee et al., 2010; Liu et al., 2010). Once the phagocyte has reached the target a direct cell membrane contact is established via a receptor-ligand interaction and phagocytosis takes place.

### Eat-me

**Figure 2:**
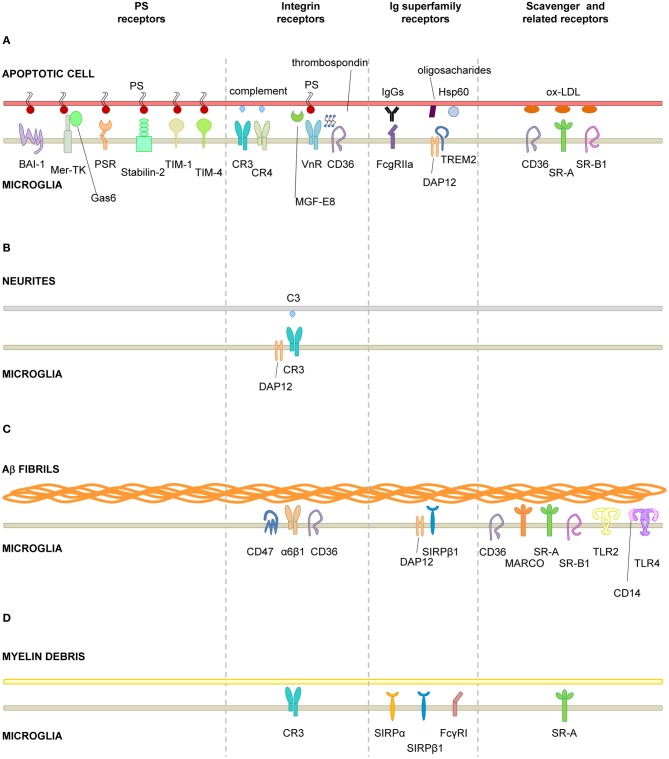
**Receptors involved in microglial phagocytosis of endogenous structures. (A)**, Apoptotic cells; **(B)**, Neurites; **(C)**, Aβ aggregates/fibrils; **(D)**, Myelin debris. Receptors have been classified into major functional/structural groups: phosphatidylserine (PS) receptors, integrin receptors, Ig superfamily-receptors, and scavenger and related receptors. For more details on the receptors, refer to Table 1. For receptors involved in the recognition of necrotic cells and microbes, refer to the main text. Abbreviations: Aβ, β-amyloid protein; BAI-1, brain angiogenesis inhibitor 1; CR, complement receptor; DAP12, DNAX-activation protein of 12 KDa; Hsp60, heat-shock protein of 60 KDa; MARCO, macrophage receptor with collagenous structure; Mer-TK, Mer tyrosine kinase; MFG-E8, milk fat globule-epidermal growth factor; Ox-LDL, oxidized low density lipoprotein; PS, phosphatidylserine; PSR, phosphatidylserine receptor; SIRPβ1, signal regulatory protein β1; SR-A, scavenger receptor class A; SR-B1, scavenger receptor class B1; TIM, T-cell-immunoglobulin-mucin; TLR, toll-like receptor; TREM2, triggering receptor expressed on myeloid cells 2; VnR, vitronectin receptor.

The process of recognition and engulfment of the targets is, arguably, the most important step in phagocytosis and where most research is concentrated. Phagocytes are equipped with a likely complementary array of receptors which enable them to recognize their targets (the so-called “eat-me” signals) and discriminate them from the remaining parenchyma, particularly from living cells (which express “don't-eat-me” signals) (Savill et al., 2002; Ravichandran, 2010). Some of these receptors serve to tether the phagocyte and the target together; others actually trigger internalization (Underhill and Goodridge, 2012). These receptors can be classified into two main types depending on their targets. Detection of pathogen-associated molecular patterns (PAMPS) is mediated through scavenger receptors in conjunction with Toll-like receptors (TLRs) such as the CD14/TLR4 complex, or receptors of the immunoglobulin superfamily (e.g., c-type lectins). Detection of apoptotic cells-associated cellular patterns (ACAMPs), of which exposure of phosphatidylserine (PS) in the outer leaflet of the cell membrane is the main exponent, is mediated directly by several receptors, including brain-specific angiogenesis inhibitor 1 [BAI-1; reviewed in Armstrong and Ravichandran (2011)], and by bridging molecules such as milk fat globule-epidermal growth factor (MFG-E8). Another important receptor that signals internalization is triggering receptor expressed on myeloid cells-2 (TREM2), whose loss of function prevents microglial phagocytosis (Takahashi et al., 2005; Hsieh et al., 2009). TREM2 is known to interact with the signaling adapter protein named DNAX-activation protein of 12 kD (DAP12) (Paloneva et al., 2002), however its ligand in apoptotic cells remains elusive. Several candidates have been proposed, namely, anionic oligosaccharides such as bacterial lipopolysaccharides (Daws et al., 2003; Quan et al., 2008) and heat shock protein 60 (Hsp60) (Stefano et al., 2009). Hsp60 is exposed in the surface of apoptotic cells (Goh et al., 2011) and increases the phagocytic activity of microglia (Stefano et al., 2009). In addition to the direct recognition of the targets by microglial cell membrane receptors, engulfment can also triggered by soluble opsonins that bind to receptors signaling internalization in the microglia. Antibodies such as IgG and proteins of the complement system such as C3b, bind to phagocyte Fc receptors and complement receptor 3 (CR3), respectively, and mediate phagocytosis (Underhill and Goodridge, 2012). Further examples of receptors and their targets are summarized in Table 1 and Figure 2 and will be provided in the following sections. The level of expression of the receptors involved in phagocytosis may change under different stimuli such as inflammation (Falsig et al., 2008), but it is not known whether they ultimately impact the efficiency of microglial phagocytosis.

**Table 1:** **Summary of receptors involved in macrophage and microglial phagocytosis**.

**Receptors**	**Ligands**	**Macrophages function**	**References**	**Microglia function**	**References**
**PS RECEPTORS**					
BAI-1	PS	Phagocytosis of apoptotic cells	Flannagan et al., 2012; Kim et al., 2012	Phagocytosis of apoptotic cells	Park et al., 2008
MER	PS [Gas 6, protein S]	Phagocytosis of apoptotic cells	Ravichandran, 2010	Phagocytosis of apoptotic cells	Grommes et al., 2008
PSR	PS	Phagocytosis of apoptotic cells	Taylor et al., 2005	Phagocytosis of apoptotic cells	De Simone et al., 2003
Stabilin-2	PS	Phagocytosis of apoptotic cells	Ravichandran, 2010; Flannagan et al., 2012	Function in phagocytosis unreported	N/R
TIM-1	PS	Phagocytosis of apoptotic cells	Flannagan et al., 2012	Phagocytosis of apoptotic cells	Noda and Suzumura, 2012
TIM-4	PS	Phagocytosis of apoptotic cells	Freeman et al., 2010; Flannagan et al., 2012	Phagocytosis of apoptotic cells	Mizuno, 2012; Noda and Suzumura, 2012
**INTEGRIN RECEPTORS**					
avβ5	PS, vitronectin [MFG-E8, thrombospondin]	Phagocytosis of apoptotic cells	Dupuy and Caron, 2008	Expressed. Function in phagocytosis unreported	Welser-Alves et al., 2011
CR1	MBL, C1q, C4b, C3b, C3bi	Adhesion to bacteria/pathogens	Fallman et al., 1993; Flannagan et al., 2012	Adhesion to opsonized erythrocytes	Ulvestad et al., 1994; Linnartz and Neumann, 2012
CR3	C3 and C1q [DAP12]	Adhesion to opsonized yeast particles; phagocytosis of bacteria; opsonized apoptotic cells; degenerated myelin and neurites	Fallman et al., 1993; Rotshenker, 2009; Linnartz and Neumann, 2012	Adhesion to opsonized yeast particles; phagocytosis of bacteria; opsonized apoptotic cells; degenerated myelin and neurites	Rotshenker, 2009; Linnartz and Neumann, 2012; Schafer et al., 2012
CR4	iC3b	Phagocytosis of opsonized apoptotic cells	Flannagan et al., 2012	Phagocytosis of opsonized apoptotic cells	Crehan et al., 2012
VnR	PS, vitronectin [MFG-E8, thrombospondin]	Adhesion to apoptotic cells; phagocytosis of apoptotic cells	Dupuy and Caron, 2008	Phagoptosis (killing of viable neurons)	Neher et al., 2011; Welser-Alves et al., 2011
**IG SUPERFAMILY RECEPTORS**					
FcγRI	IgG1 = IgG3 > IgG4	Phagocytosis of degenerated myelin	Rotshenker, 2009	Phagocytosis of degenerated myelin	Noda and Suzumura, 2012
FcγRIIa	IgG3 ≥ IgG1 = IgG2	Phagocytosis of pathogens and apoptotic cells	Hart et al., 2004	Phagocytosis of pathogens and apoptotic cells	Linnartz and Neumann, 2012
RAGE	Aβ, AGEs, PS, and HMGB1, C1q	Phagocytosis of apoptotic cells	He et al., 2011	Mediates pro-inflammatory effect of Aβ	Block et al., 2007; Noda and Suzumura, 2012
Siglec11	α 2,8-linked polysialic acids	Reduced phagocytosis of apoptotic cells	Linnartz and Neumann, 2012	Reduced phagocytosis of apoptotic cells	Wang and Neumann, 2010; Linnartz and Neumann, 2012
SIRPα	Myelin [SP-A, D; CD47]	Recognition and downregulation of phagocytosis of myelin	Linnartz and Neumann, 2012	Recognition and downregulation of phagocytosis of myelin	Ransohoff and Perry, 2009; Noda and Suzumura, 2012
SIRPβ1	Unknown ligand [DAP12]	Increase of phagocytosis of opsonized red blood cells	Hayashi et al., 2004; Gaikwad et al., 2009	Phagocytosis of neuronal debris, fibrillary Aβ, latex beads	Gaikwad et al., 2009; Linnartz and Neumann, 2012
TREM2	Hsp60, oligosaccharides [DAP12]	Phagocytosis of apoptotic cells	Klesney-Tait et al., 2006	Phagocytosis of apoptotic cells	Klesney-Tait et al., 2006; Ransohoff and Perry, 2009
**SCAVENGER AND RELATED RECEPTORS**					
CD36	Ox-LDL, Ox-PS [thrombospondin]	Adhesion to apoptotic cells; phagocytosis of apoptotic cells	Greenberg et al., 2006; Flannagan et al., 2012	Phagocytosis of apoptotic cells	Noda and Suzumura, 2012
CD68	Ox-LDL	Adhesion to erythrocytes	Hoffmann et al., 2001	Phagocytic marker. Function in phagocytosis unreported	Fulci et al., 2007
LOX-1	LDL, Ox-LDL, Hsp70	Phagocytosis of aged/apoptotic cells	Taylor et al., 2005	Expressed. Pro-inflammatory response. Function in phagocytosis unreported	Zhang et al., 2012
MARCO	Ac-LDL, bacteria	Adhesion to unopsonized particles and bacteria; phagocytosis of bacteria, apoptotic cells, and unopsonized latex beads	van der Laan et al., 1999; Rogers et al., 2009	Decrease of antigen internalization capacity; adhesion to Aβ, bacteria; decreased bead phagocytosis	Granucci et al., 2003; Block et al., 2007
SR-A	LPS, lipotheicoic acid, Ac-LDL	Adhesion to apoptotic thymocytes; phagocytosis of bacteria, apoptotic cells, and degenerated myelin	Savill et al., 2002; Taylor et al., 2005; Flannagan et al., 2012	Phagocytosis of bacteria, apoptotic cells, degenerated myelin and Aβ	Block et al., 2007; Wilkinson and El Khoury, 2012
SR-B1	HDL, LDL, Ox-HDL, Ox-LDL, advanced glycosylation end products	Phagocytosis of bacteria and apoptotic cells	Boullier et al., 2001	Adhesion to Aβ, phagocytosis of apoptotic cells, and endocytosis of fibrillar Aβ	Block et al., 2007
TLR2	Pam3Cys/Glycolipids, Hsp70, HMGB1	Function in phagocytosis unreported	N/R	Phagocytosis of Aβ	Landreth and Reed-Geaghan, 2009; Noda and Suzumura, 2012
TLR4	LPS, lipotheicoic acid, Hsp60,70; co-receptor CD14	Phagocytosis of bacteria	Anand et al., 2007; McCoy and O'Neill, 2008	Bacterial recognition; phagocytosis of Aβ	Block et al., 2007; Noda and Suzumura, 2012
**C-TYPE LECTIN RECEPTORS**					
Dectin-1	β glucans	Phagocytosis of yeast, fungus	Lowell, 2011; Flannagan et al., 2012	Phagocytosis of yeast, fungus	Shah et al., 2008
MR	Mannose, fucose	Phagocytosis of pathogens	Flannagan et al., 2012	Phagocytosis of yeast	Marzolo et al., 1999; Zimmer et al., 2003
**OTHER RECEPTORS**					
β2-GPI receptor (unidentified)	PS [β2-GPI]	Phagocytosis of apoptotic cells	Lauber et al., 2004; Taylor et al., 2005	Function in phagocytosis unreported	N/R
CD91	Multiprotein complex (calreticulin, MBL, C1q), HSPs	Initiate engulfment of apoptotic cells	Ogden et al., 2001	Function in phagocytosis unreported	Pais et al., 2008

*Receptors have been classified into major functional/structural groups: PS receptors, integrin receptors, Ig superfamily receptors, scavenger and related receptors, C-type lectin receptors, and others. Bridging molecules are indicated in brackets*.

Abbreviations *Aβ* *amyloid beta protein* *Ac-LDL* *acetylated low density lipoprotein* *AGEs* *advanced glycation end products* *BAI-1* *brain angiogenesis inhibitor 1* *CR* *complement receptor* *DAP12* *DNAX-activation protein of 12 KDa* *HDL* *high density lipoprotein* *HMGB1* *high-mobility group box 1 protein* *Hsp* *heat-shock protein* *LDL* *low density lipoprotein* *LOX-1* *lectin-like oxidized LDL receptor-1* *LPS* *bacterial lipopolysaccharides* *MAC-1* *macrophage antigen complex 1* *MARCO* *macrophage receptor with collagenous structure* *MBL* *mannan-binding lectin* *Mer-TK* *Mer tyrosine kinase* *MFG-E8* *milk fat globule-epidermal growth factor* *MR* *mannose receptor* *N/R* *non-reported* *Ox-HDL* *high density lipoprotein* *Ox-LDL* *oxidized low density lipoprotein* *Ox-PS* *oxidized phosphatydilserine* *PS* *phosphatydilserine* *PSR* *phosphatydilserine receptor* *RAGE* *receptor for advanced glycation endproducts* *Siglec11* *sialic acid binding immunoglobulin-like lectin 11* *SIRPα* *signal regulatory protein alpha* *SIRPβ1* *signal regulatory protein beta 1* *SP* *surfactant protein* *SR* *scavenger receptor* *TIM* *T-cell-immunoglobulin-mucin* *TLR* *toll-like receptor* *TREM2* *triggering receptor expressed on myeloid cells 2* *VnR* *vitronectin receptor.*

These receptors and their targets closely interact in what has been termed “engulfment synapse” or “phagocytic synapse” (Ravichandran, 2010; Dustin, 2012; Underhill and Goodridge, 2012). Similar in size (0.5 μm in diameter) and purpose (close cell–cell contact) to its immunological and neural counterparts, phagocytic synapses are specialized regions of the membrane where the apoptotic cell and the phagocyte interact through microclusters of receptors. Like the immunological synapse formed between antigen presenting cells (APCs) and T lymphocytes, and in contrast to the synapses formed between neurons, phagocytic synapses are short-lived and last only a few minutes (Dustin, 2012). Phagocytic synapses are also characterized by exclusion of phosphatases (Dustin, 2012), so that once the contact is established the signal is transduced by a variety of intracellular kinases (Syk kinase, phosphatidylinositol 3-kinase) and small GTPases (Rac, Rho) and adapter molecules such as engulfing and cell motility (ELMO) family (Gumienny et al., 2001; Lee et al., 2007). An emerging pivotal complex is that formed by ELMO-1 and dedicator of cytokinesis 180 (DOCK-180), which is activated among other receptors by BAI-1 and act as a guanine nucleotide exchange factor for Rac (Park et al., 2008; Patel et al., 2011). ELMO-1 function is regulated by phosphorylation by the hematopoietic cell kinase (HCK) (Yokoyama et al., 2005), a member of the Src family whose deficiency impairs macrophage phagocytosis (Lowell et al., 1994). These pathways lead to the remodeling of the phagocyte cytoskeleton through actin polymerization and membrane composition, triggering the formation of pseudopodia that form a phagocytic cup engulfing the target (Lee et al., 2007). Of the complex process of signal transduction and formation of the phagocytic cup, very little is known in microglia.

### Digest-me

The phagocytic cup closes and thus forms the phagosome around the target. To execute the degradation of the target, phagosomes go through a process of maturation in which they fuse sequentially with early and late endosomes, and lysosomes, to form phagolysosomes (Desjardins et al., 1994). These phagolysosomes contain hundreds of proteins, including hydrolases such as cathepsins to digest the target; and proton pumps such as vacuolar ATPases to acidify the medium (Garin et al., 2001). The acidic pH found in phagolysosomes (pH ≤ 5) is essential for the lysosomal degradation capabilities, as it is optimal for most hydrolases. Interestingly, the signaling associated with the acidic pH of lysosomes deactivates the generation of radicals from the oxidative burst (Li et al., 2010). Again, the process of phagosome formation and cargo degradation has been barely addressed in microglia. A recent study in *C. elegans* using live imaging showed that in this nematode microglial vATPases are required for phagosome–lysosome fusions and consequently to degrade cargo (Peri and Nusslein-Volhard, 2008). Further research is necessary to delineate the mechanisms of degradation of structurally different cargo, ranging from Aβ deposits to whole apoptotic cells, in microglia. Moreover, because phagocytosis is not only performed by ameboid, activated cells but also by terminal or en passant branches of ramified, resting microglia (Peri and Nusslein-Volhard, 2008; Sierra et al., 2010), the location where the actual degradation occur remains unexplored. Live imaging experiments show that resembling phagosomal cups are retrogradely transported to the cell soma in the mouse neocortex (Nimmerjahn et al., 2005); small puncta of apoptotic cell material are observed within branches of ramified microglia in fixed adult hippocampus, indirectly suggesting their transport (Sierra et al., 2010). Direct evidence of retrograde transport of cargo-containing phagosomes was recently found in *C. elegans* (Peri and Nusslein-Volhard, 2008). Collectively, these data indirectly suggest a yet unexplored role of the cytoskeleton in transporting the phagosome for the degradation of the engulfed material.

Overall, phagocytosis is considered to be essential for maintaining brain tissue homeostasis. The rapid clearance of apoptotic cells prevents their transformation into secondary necrotic cells (as it readily occurs *in vitro*), with the subsequent loss of permeability of the cell membranes and spillover of intracellular contents. For instance, the phagocytosis of apoptotic neutrophils and T cells during the resolution of the inflammatory response prevents the release of intracellular granules containing lysosomal enzymes and possibly acidification of the extracellular space (Magnus et al., 2001). Further, blockade of microglial phagocytosis of polymorphonuclear neutrophils, which infiltrate the brain parenchyma after focal ischemia, decreases neuronal viability in organotypic slices (Neumann et al., 2008). Phagocytosis of apoptotic neurons is also beneficial for neuronal survival. For instance, neurons can be rescued from excitotoxic death when phagocytosis is promoted by the chemokine fractalkine in neuron-microglia co-cultures, but not in neuronal cultures alone (Noda et al., 2011). In addition, myelin debris contains inhibitory molecules which inhibit axonal regeneration (Gitik et al., 2011); Aβ plaques cause physical damage of the neural tissue and lead to functional deficits if they are not removed (Wang et al., 2011); and removal of axonal debris by microglia increases axonal regeneration *in vitro* (Tanaka et al., 2009; Hosmane et al., 2012).

In the living brain, however, the beneficial effects of microglial phagocytosis have been harder to prove, possibly due to the lack of molecular tools to specifically interfere with microglial phagocytosis. Enhancing brain phagocytosis via intravenous delivery of TREM2-transduced bone marrow-derived precursor cells potentiates the clearance of degenerated myelin, decreases the inflammatory response and ameliorates the course of the disease in the mouse model of multiple sclerosis (MS), experimental acute encephalomyelitis (EAE) (Takahashi et al., 2007). A method recently proposed is the use of annexin V, a PS binding protein, to block phagocytosis. When administered systemically, annexin V reaches the brain and, as expected, leads to accumulation of apoptotic debris, although it has not been quantified whether it actually prevents phagocytosis (Lu et al., 2011). Treatment with annexin V impaires the generation of newborn neurons in adult neurogenic niches without affecting the proliferation of neuronal precursors, suggesting that phagocytosis is important for the differentiation and/or survival of the newborn cells (Lu et al., 2011). Direct evidence of the beneficial effects of microglial phagocytosis was shown recently in a mouse model of Rett syndrome by knocking out the methyl-CpG-binding protein Mecp2 (Derecki et al., 2012). While Rett syndrome is mostly considered a primary neuronal disease, defects in phagocytosis and inflammatory response are found in Mecp2-deficient microglia (Derecki et al., 2012). Further, bone marrow cells transplanted into irradiated Rett mice differentiate into microglia and partially arrest the pathology of the disease (Derecki et al., 2012). Interestingly, the disease is mimicked by microglial-specific depletion of Mecp2 and phagocytosis is blocked by intravenous injection of annexin V, but not by either manipulation independently. These data suggest that a failure in microglial phagocytosis does not underlie the pathology of the Rett syndrome but, nonetheless, increasing phagocytosis may be beneficial (Derecki et al., 2012).

## Functional consequences of phagocytosis

Clearance of microbes or debris is a direct intervention of microglia to remove invading pathogens or to clean up the tissue from unwanted material. The direct consequences of this clearance function were already described in the chapter above. Furthermore, ingestion and degradation of targets has several indirect consequences for the phagocyte as well as the surrounding tissue. Phagocytosis triggers activation of several intracellular signaling pathways and remodels the cytoskeleton and cell membrane (Lee et al., 2007). In addition, microglia receive a tremendous metabolic load from the lipids, carbohydrates, proteins, and other components from the digested target (particularly in the case of apoptotic cells and microorganisms), leading to direct changes in the phagocyte's lipid and cholesterol, and possibly glucose metabolism (Han and Ravichandran, 2011). In addition, whether phagocytosis impacts proliferation, survival, or differentiation of the phagocyte remains unexplored. In contrast, three major functional consequences of microglial phagocytosis have been well-described: antigen presentation, activation of the respiratory burst, and modulation of inflammatory responses.

### Antigen presentation

Professional APCs (macrophages, DCs, and B lymphocytes) engulf their targets locally; next, they travel to lymph nodes, where the digested exogenous proteins (antigens) are expressed in their membrane attached to specific receptors (major histocompatibility complex class II, or MHC-II), which enable their recognition by naïve T lymphocytes, initiating the cellular arm of the adaptive immune response. Antigen presentation is a complex process beyond the scope of this review. Nonetheless, it is important to note that antigen presentation in the CNS has been a matter of hot debate in the last few years [reviewed by Ransohoff and Engelhardt (2012)]. In normal conditions, microglia do not express MHC-II and only a subpopulation expresses co-stimulatory molecules such as CD11c (Bulloch et al., 2008), and in agreement they have poor antigen presenting activity *in vivo* (Ford et al., 1995). Upon local inflammatory challenge, microglia show increased expression of MHC-II and CD11c *in vivo* and increased antigen presentation *in vitro*, albeit with much lower efficiency than professional APCs (Gottfried-Blackmore et al., 2009). While it is clear that they have the capacity of presenting antigens under some particular conditions, the microglial contribution to antigen presentation *in vivo* is thought to be mostly irrelevant (Galea et al., 2007; Ransohoff and Engelhardt, 2012). A major unresolved issue is whether microglia would have the capability of abandoning the brain parenchyma and reach the lymph nodes. In fact, antigen presentation in the CNS is thought to occur not in the parenchyma, but in the meninges and choroid plexus, which contain perivascular APCs, or by direct drainage of CNS antigens in the cerebrospinal fluid from the subarachnoidal space to channels in the cribiform plate and ultimately into deep cervical lymph nodes (Galea et al., 2007; Ransohoff and Engelhardt, 2012). Self-antigens, including CNS antigens, do not produce a response since self-reactive T cells are eliminated by clonal deletion in the thymus. In the autoimmune disease MS, however, myelin-specific T cells may escape from this tolerance mechanism and enter the brain under some circumstances, leading to demyelination (Goverman, 2011). Microglia seems to play a negative role in the disease progression of EAE, the animal model of MS, as its depletion reduces the severity of the disease (Heppner et al., 2005). Whether this potentially detrimental role of microglia in EAE is related to phagocytosis and/or antigen presentation remains unknown (Ransohoff and Engelhardt, 2012).

### Respiratory burst

During phagosome maturation the enzyme nicotinamide adenine dinucleotide phosphate (NADPH) oxidase is assembled in the phagosome. NADPH oxidase catalyzes the reaction of NADPH and oxygen to form NADP^+^, protons and the superoxide anion (O_2^−^_), in a process known as the respiratory burst (Minakami and Sumimotoa, 2006). In the acidic pH of the phagosome, the superoxide anion is dismutated into hydrogen peroxide, H_2_O_2_, and later transformed into other reactive oxygen species (ROS) which contribute to killing of engulfed microorganisms and degradation of other cargo (Minakami and Sumimotoa, 2006). ROS are extremely aggressive oxidants and can induce both apoptosis and necrosis (Pourova et al., 2010). Potentially toxic for the phagocyte itself and the surrounding tissue, they are thought to be released exclusively within the phagosome. In fact, NADPH assembly is a tightly controlled process in phagocytes and includes the recruitment to the phagosome of the catalytic core gp92^phox^ and p22^phox^, and the phosphorylation and membrane translocation of the regulatory subunit p47^phox^ (Quinn and Gauss, 2004). To ensure further regulation, the enzyme is rapidly deactivated, resulting in transient activity (Decoursey and Ligeti, 2005). Most of our understanding about the molecular mechanisms involved in activation or the respiratory burst comes from studies performed in neutrophils (Minakami and Sumimotoa, 2006), one of the most important components of the immune system in the defense against invading microorganisms. In microglia, the respiratory burst appears to be activated by phagocytosis through similar mechanisms (Ueyama et al., 2004) after phagocytosis of zymosan (a yeast cell wall preparation) (Newell et al., 2007) or myelin (Williams et al., 1994), although it remains to be studied whether it is also activated after phagocytosis of other types of cargo.

Recently, it has been suggested that the NADPH oxidase can be assembled in alternative locations, driving the respiratory burst in the absence of phagocytosis (Bylund et al., 2010). In microglia, the respiratory burst is triggered by hypoxia/reoxygenation (Spranger et al., 1998) and LPS (Qin et al., 2004) and results in the extracellular release of ROS. In turn, inflammation-induced respiratory burst leads to the release of glutamate by microglia, further contributing to neuronal damage (Barger et al., 2007). Interestingly, the microglial respiratory burst has been linked to induction of neuronal apoptosis in the developing cerebellum *in vitro* (Marin-Teva et al., 2004) and in the developing hippocampus *in vivo* (Wakselman et al., 2008). In both studies, ROS-trapping by free radical scavengers resulted in a significant rescue of neurons from apoptosis. However, it remains unclear whether the activation of the respiratory burst was activated as a consequence of phagocytosis or by independent mechanisms. While microglia were clearly identified as the only source of ROS, the actual intracellular location (i.e., in the phagolysosome or otherwise) was not addressed (Marin-Teva et al., 2004; Wakselman et al., 2008). The hippocampal study suggested indirectly that phagocytosis was indeed involved in the activation of the respiratory burst, because mice lacking the complement receptor CR3 or expressing mutant DAP12 showed reduced ROS production and apoptosis (Wakselman et al., 2008). In spite of the pro-phagocytic role of CR3 and DAP12, whether phagocytosis was disrupted in these transgenic mice was not addressed. Two possible scenarios remain open: in the first one, microglia would attack live (possibly healthy) neurons, phagocytose them via CR3/DAP12, and kill them intracellularly through the respiratory burst. In the second scenario, an undetermined stimulus would activate microglia to produce ROS via CR3/DAP12, which would be released extracellularly and kill neurons, subsequently phagocytosed by microglia. Clearly, more precise methods to quantify ROS (extracellular, intracellular, phagolysosomal, etc.) (Bylund et al., 2010) and to quantify phagocytosis and the nature of the cargo (Sierra et al., 2010) are necessary to unravel the role of microglial phagocytosis and respiratory burst in the developing hippocampus and cerebellum.

### Modulation of inflammatory responses

Historically, it was believed that phagocytosis of apoptotic cells was a neutral immune event because it does not initiate an inflammatory response [reviewed by Savill et al. (2002)]. Over the past 20 years, it has been made clear that the phagocytosis of apoptotic cells is largely anti-inflammatory (Stern et al., 1996; Voll et al., 1997; Fadok et al., 1998). The inhibition of pro-inflammatory cytokines synthesis requires tethering of the phagocyte and the apoptotic cell, but not actual engulfment, followed by paracrinal release of the anti-inflammatory cytokine transforming growth factor beta (TGFβ), although the mechanism is not well-understood (Lucas et al., 2006). In addition, phagocytosis of other types of cargo such as myelin can trigger an anti-inflammatory response (Liu et al., 2006). On the contrary, phagocytosis of microbes associated with TLR stimulation is pro-inflammatory (Erdman et al., 2009). The anti-phlogistic response to phagocytosis of apoptotic cells also occurs in microglia. Microglia phagocytosing apoptotic T cells produce less tumor necrosis factor alpha (TNFα), a major pro-inflammatory cytokine, than naïve microglia after LPS challenge *in vitro* (Magnus et al., 2001). Similarly, phagocytosis of an apoptotic neuronal line induces an increased production of TGFβ and neural growth factor (NGF) in basal conditions; as well as a decreased production of TNFα, nitrite, and prostaglandin E2 during LPS challenge *in vitro* (De Simone et al., 2003). These anti-inflammatory effects are mediated by microglial PS receptor (De Simone et al., 2002), as well as by TREM2 (Takahashi et al., 2005). More recently it has been described that the immunosuppression induced by phagocytosis of apoptotic cells depends on the presence of the complement protein C1q bound to apoptotic cells, without which phagocytosis is pro-inflammatory (Fraser et al., 2010). It is important to note that complement proteins are present in the fetal bovine serum commonly used in primary cultures. Nonetheless, it remains to be determined whether the opsonin C1q is produced in the normal brain and binds to apoptotic cells phagocytosed by microglia *in vivo*. As a matter of fact, there is a big gap in our knowledge of the type of inflammatory response resulting from phagocytosis *in vivo*. The only *in vivo* data derives from a model of prion disease, where phagocytic microglia have been qualitatively observed not to express the pro-inflammatory cytokine interleukin 1beta (IL-1β) (Hughes et al., 2010). Because inflammation has severe effects in the brain, including neurotoxicity (Pickering et al., 2005) and epileptogenesis (Galic et al., 2012), more research is necessary to conclusively determine whether microglial phagocytosis of apoptotic cells is anti-inflammatory *in vivo*.

## Microglial phagocytosis in physiological conditions

**Figure 3:**
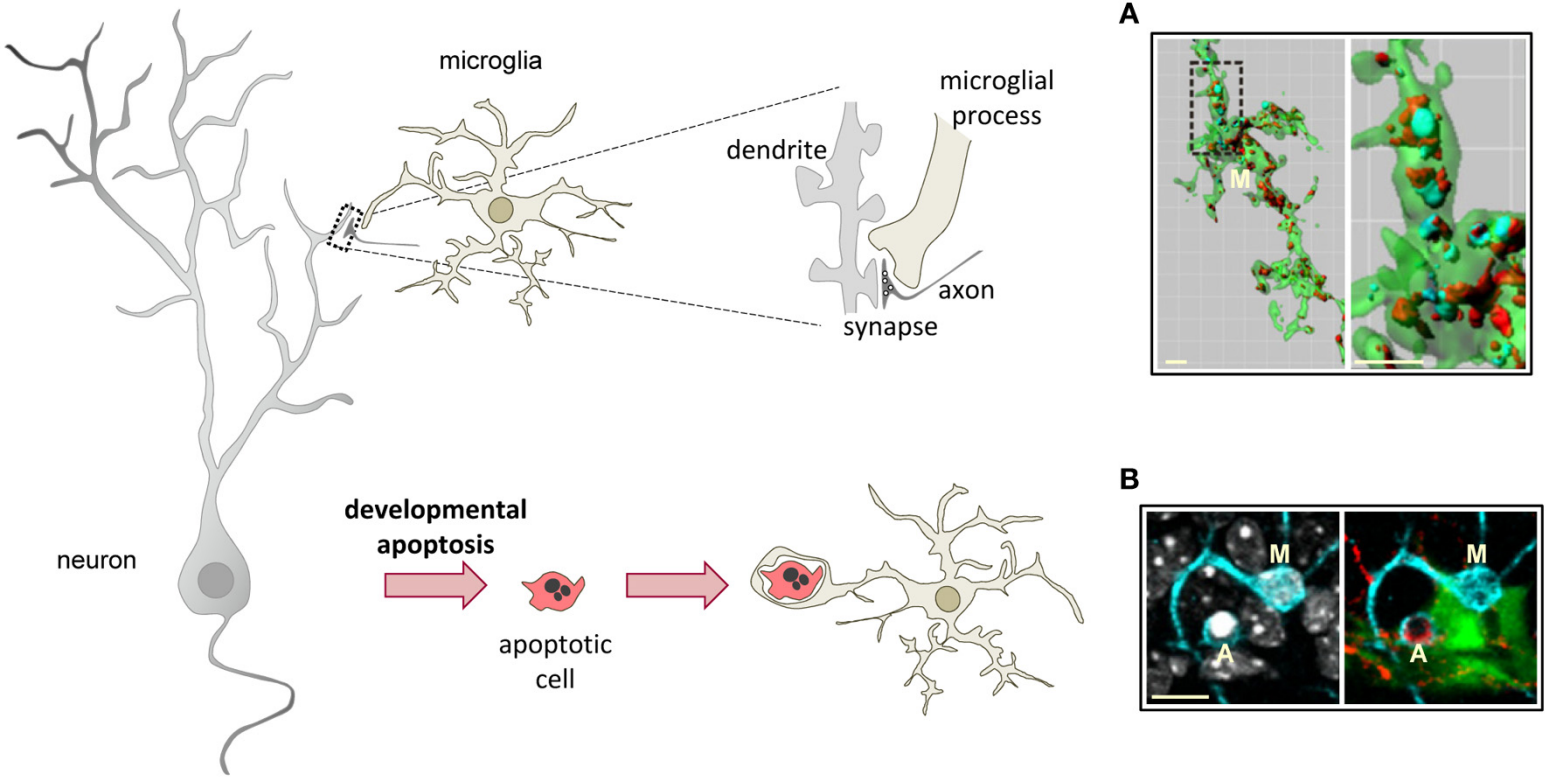
**Microglial phagocytosis in health.** Motile, ramified, unchallenged microglia phagocytose neurites (**A**, dendritic spines, axons) and developmentally apoptotic neurons **(B)** in physiological conditions in the adult and developing brain. **(A)** Surface-rendered CX3CR1^+^/EGFP microglia (M; green) from the dorsolateral geniculate nucleus engulfing ipsi- (blue, labeled with cholera-toxin **(B)** conjugated to Alexa 647) and contralateral (red, labeled labeled with cholera-toxin **(B)** conjugated to Alexa 594) inputs from retinal ganglion cells at postnatal day 5, when robust pruning occurs. The insert is shown at higher magnification on the right panel. **(B)** Iba-1 labeled microglia (M; cyan) branching a phagocytic pouch which engulfs a newborn apoptotic/pycnotic cell (A; labeled with the DNA dye DAPI, white), containing immunoreactivity for the neuroblast marker polysialic acid neural cell adhesion molecule (red). The cell is located in the subgranular zone of the hippocampus, where neural stem cells (visualized in nestin-GFP mice) are located and proliferate throughout adulthood. Images **(A)** and **(B)** are reprinted with permission from Elsevier. **(A)** is reprinted from Schafer et al. (2012). **(B)** is reprinted from Sierra et al. (2010).

The widely accepted theory of linear activation of microglia proposed by Gennadij Raivitch, William Streit, and collaborators (Raivich et al., 1999; Streit et al., 1999) leads to the generalized assumption that microglia have to be preactivated or primed by inflammatory challenge in order to become efficient phagocytes (Kettenmann, 2007). More current are the terms M1 and M2 to describe a spectrum of states of microglial activation, similar to the polarization of macrophages responses (Mantovani et al., 2002). The M1 phenotype or classic activation, is induced by PAMPs and pro-inflammatory cytokines, and is characterized by high expression of TLRs, TNFα, co-regulatory molecules for antigen presentation, and increased release of ROS. On the contrary, the M2 phenotype, or alternative activation, is driven by stimuli such as interleukin 4 (IL-4) or interleukin 13 (IL-13), and is characterized by the production of anti-inflammatory interleukin 10 (IL-10) and TGFβ, and higher expression of scavenger receptors. The M1 phenotype is basically neurotoxic, while the M2 phenotype is more neuroprotective (Durafourt et al., 2012). However it is not very clear what is the relationship between the M1/M2 phenotypes and phagocytosis. For instance, classic activation paradigms driving a M1 phenotype lead to increased enhanced engulfment of apoptotic cells *in vitro* (Chan et al., 2001; McArthur et al., 2010). In addition, expression of phagocytic receptors is triggered by many inflammatory and neurodegenerative paradigms *in vivo* assumedly inducing an M1 phenotype. On the other hand, a M2 phenotype driven by IL-4 and IL-13 increases the phagocytosis of myelin (Durafourt et al., 2012), whereas a M2 phenotype induced by glioma cancer stem cells reduces phagocytosis (Wu et al., 2010). Regardless of the nomenclature used to describe microglial phenotype after activation, the notion that microglia require to be preactivated to phagocytose efficiently is challenged by a number of recent papers showing that “resting” or “steady-state” microglia are efficient phagocytes of spines and apoptotic cells during physiological conditions in the adult and developing brain (Dalmau et al., 2003; Sierra et al., 2010; Schafer et al., 2012) (Figure 3).

### Microglial phagocytosis of spines

The capacity for microglia to phagocytose synapses has just begun to be unraveled. In the juvenile visual cortex, most microglia interact with synaptic elements (spines, axon terminals, perisynaptic astrocytes, and synaptic clefts) (Tremblay et al., 2010). Furthermore, live imaging indirectly supported a role for microglia in pruning synapses, as spines contacted by microglia where more frequently eliminated than non-contacted spines (Tremblay et al., 2010). Qualitative examples of microglia containing pre- and postsynaptic material labeled respectively with SNAP25, a protein associated with presynaptic vesicles, and with PSD95, a marker of the excitatory postsynaptic density, have been shown in the developing postnatal hippocampus (Paolicelli et al., 2011). Interestingly, a transient increase in dendritic spines and immature synapses was observed in CX3CR1 knock-out mice compared to wild-type mice, possibly due to a reduction in microglial density (Paolicelli et al., 2011). Unfortunately, the authors did not carry out a quantification of microglial phagocytosis of spines in CX3CR1-KO mice and thus the role of the fractalkine receptor as an opsonin for microglial phagocytosis of spines cannot be ruled out. Direct, quantified evidence of microglia phagocytosing synapses (mostly, presynaptic elements) in the developing visual system has been recently provided using confocal and electron microscopy (Schafer et al., 2012). In the dorsal lateral geniculate nucleus (dLGN), active pruning of synaptic inputs from the retinal ganglion cells (RGCs) produces well-established ipsi- and contralateral innervations territories. This pruning is at least partially mediated by complement-mediated microglial phagocytosis, because mice deficient either for the receptor CR3 or its ligand, C3, have decreased phagocytosis of synaptic inputs, a sustained increase in synaptic density, and deficits in segregation of the eye-specific territories compared to wild-type mice (Schafer et al., 2012). Thus, unwanted synapses seem to be tagged for removal by deposition of complement proteins but—who is the decision maker? Do microglia actively instruct which synapses must be removed? *In vitro*, microglia produce several components of the complement cascade, including C1q and C3, which are involved in the phagocytosis of neurites via CR3 (Linnartz et al., 2012). Interestingly, removal of the monosaccharide sialic acid from the neural glycocalyx is essential for C1q binding to neurites and subsequent microglial phagocytosis in cultured neurons (Linnartz et al., 2012). It seems plausible that synapse pruning is regulated by a combination of mechanisms involving activity-dependent control of synaptic strength, alterations in the neuronal glycocalyx, and tagging of altered synapses by microglial-release of complement proteins, followed by removal of pre- and/or postsynaptic components by microglial phagocytosis. In contrast to the more established mechanism of synaptic pruning by simple retraction or degeneration of the input axon (Luo and O'Leary, 2005), microglia-dependent pruning is likely to require more time and energy, while perhaps adding more levels of control to a phenomenon which is essential to determine brain connectivity. A possible similar combination of mechanisms has been observed in the developmental pruning of dendrites in Drosophila neurons, where branch retraction and local fragmentation of dendrites are observed together with phagocytosis of caspase-labeled dendrites by blood phagocytes (Williams and Truman, 2005; Williams et al., 2006).

Overall, these data support a novel role for microglia in monitoring synapses in the healthy developing brain, with potentially profound consequences for diseases in which neural connectivity and microglial activation concur. One such disease is transient ischemia, in which the turnover of synapses (Zhang et al., 2005) as well as microglial activation and inflammation increase (Lambertsen et al., 2012). In the ischemic cerebral cortex microglial contacts with synaptic boutons last-longer (1 h) than in the control cortex (5 min) (Wake et al., 2009). While it is tempting to speculate that extended contacts are related to synapse pruning, only a minority of boutons disappeared after being contacted by microglia and phagocytosis was not observed (Wake et al., 2009). In autism spectrum disorders (ASD), a complex set of diseases with unknown pathophysiology, both neural hyperconnectivity (Testa-Silva et al., 2012) and inflammation (Vargas et al., 2005) develop. In MECP2-deficient mice, a model of Rett syndrome, microglia has defective phagocytosis *in vitro*, and preventing phagocytosis in the mice exacerbates the symptoms (Derecki et al., 2012). The potential defects in spine and/or apoptotic cell phagocytosis by microglia in Rett syndrome and other ASDs remain to be elucidated.

### Microglial phagocytosis of apoptotic cells during brain development and in neurogenic niches

A detailed map estimating microglial phagocytosis during development showed an overall high efficiency of microglia-removing apoptotic cells (Dalmau et al., 2003). At time points where apoptosis is maximal, microglia phagocytose 97% of apoptotic cells in the fimbria (embryonic day E18), 88 and 100% in the CA region of the hippocampus (E16 and postnatal day 0, respectively), 18 and 86% in the cerebral cortex (E16 and P0, respectively), and 93% in the dentate gyrus (P0). These estimates match well with the phagocytic efficiency of microglia in the dentate gyrus throughout adulthood, where new neurons are continuously produced. From 1 to 12 months of age in mice, microglia phagocytose over 90% of the cells undergoing apoptosis (Sierra et al., 2010). Nonetheless, the phagocytic capacity is region-specific, as microglia only phagocytose 50% of the apoptotic cells at the maximum period of cell death (P3) in the developing cerebellum (Ashwell, 1990; Marin-Teva et al., 2004; Wakselman et al., 2008).

But, what happens to the remaining apoptotic cells? A possible interpretation is that they will be engulfed by microglia at a later time point, so that the percentage of non-phagocytosed apoptotic cells is an indirect representation of the “find-me” time. Alternatively, these cells may be disposed of through other mechanisms, such as phagocytosis by other cell types. Microglia are considered the professional brain phagocytes. Non-professional phagocytes such as astrocytes delay phagocytosis for several hours (Parnaik et al., 2000) and engulf with much lower capacity (Magnus et al., 2002), at least *in vitro*. Nonetheless, other cell types mediate phagocytosis of apoptotic cells *in vivo* during development. For instance, in the developing cerebellum (P7) phagocytosis of apoptotic oligodendrocytes is carried out by specialized astrocytes, the Bergmann glia, albeit at a relative low efficiency (55% of apoptotic cells engulfed) (Parnaik et al., 2000). In the developing retina, phagocytosis is executed by microglia and Müller cells (Egensperger et al., 1996), and throughout lifetime the excess of photoreceptors membrane shredded is phagocytosed by retinal pigment epithelium (RPE) cells (Kevany and Palczewski, 2010). While considered non-professional phagocytes, RPE cells have an enormous phagocytic capacity, as they engulf up to 10% of the photoreceptors volume on a daily basis (Kevany and Palczewski, 2010). In the adult hippocampal neurogenic cascade, only microglia have been observed to phagocytose apoptotic newborn cells (Sierra et al., 2010). Nonetheless, neural-committed neuroprogenitors (neuroblasts, labeled with doublecortin) have some phagocytosing capabilities which contribute to the maintenance of the neurogenic cascade, although what they actually take in remains to be determined (Lu et al., 2011). In peripheral ganglia, the apoptotic neurons are phagocytosed by satellite glial cell precursors (Wu et al., 2009). It has been speculated that low microglial density in some regions may be related to the recruitment of non-professional phagocytes (Parnaik et al., 2000) but the reasons behind this promiscuous phagocytosis during brain development are not known. Assumedly, microglial phagocytosis contributes to the maintenance of tissue homeostasis during development and in adult neurogenic niches by rapidly removing cellular debris. It can also be speculated that microglial phagocytosis takes a more active role in regulating neurogenesis. For instance, after phagocytosis, cultured microglia produce higher levels of TGFβ and NGF (De Simone et al., 2003), which are negative and positive regulators of hippocampal neurogenesis, respectively (Buckwalter et al., 2006; Frielingsdorf et al., 2007). After stroke, microglia in the subventricular zone (SVZ) show a pro-neurogenic phenotype which includes the expression of insulin-like growth factor 1 (IGF-1) (Thored et al., 2009), another well-known inducer of neurogenesis (O'Kusky et al., 2000). Stroke-responding microglia were labeled with ED1 but unfortunately phagocytosis was not quantified. Future research will delineate the contribution of microglia to neurogenesis.

The high efficiency of phagocytosis, that is, the high coupling between apoptosis and phagocytosis, has also suggested the idea that phagocytosis executes the final stages of apoptotic cell death. Apoptosis can be initiated by two major pathways: the extrinsic pathway, driven by activation of membrane receptors such as CD95, or the TNFα receptor; and the intrinsic pathway, initiated by cellular stress (DNA mutations, deprivation of survival factors, Ca^2+^ overload, etc.) (Reubold and Eschenburg, 2012). While the signaling cascades are complex and varied, a common mechanism of execution of most forms of apoptosis is the formation of the apoptosome, a macromolecular complex which activates the effector caspase 3, a cystein protease responsible for DNA fragmentation, membrane blebbing, cytoskeleton degradation, and the other major hallmarks of apoptosis (Blank and Shiloh, 2007). In addition to the cell-autonomous degradation mediated by caspases, phagocytes may also contribute to carry out death. For instance, mutations in *C. elegans* engulfment genes, such as ced-1, permit the survival of cells that would normally die (Reddien et al., 2001). In addition, DNA fragmentation is not only mediated by cell-autonomous caspase-activated DNase (CAD) but can also be partly attributed to postengulfment degradation by lysosomal enzymes (McIlroy et al., 2000). The lysosomal DNase II of macrophages contributes to thymocyte DNA degradation during thymus development (Kawane et al., 2003). Whether this mechanism is universal to macrophages and microglia is unknown, because DNase II deficient mice embryos show severe defects in the thymus and kidney, but not in the brain (Kawane et al., 2003). Nevertheless, these evidences suggest that, since there are no stop points in apoptosis analog to the check points found in mitosis, phagocytosis is the de facto mechanism to discriminate between moribund and dead cells.

In some circumstances this effective coupling may have detrimental consequences. It has been suggested that if phagocytosis is too effective, it may lead to the removal of cells which will otherwise have time to repair themselves (Kao et al., 2011). For example, the macrophages of mice and worms deficient in the secreted glycoprotein progranulin have an enhanced phagocytic efficiency, which is perhaps related to the neurodegeneration found in human patients of familial frontotemporal lobar degeneration, mostly caused by mutations in progranulin (Kao et al., 2011). Going further, microglial phagocytosis may be the primary cause of cell death under some circumstances. Macrophages are known to interact with live cells but disengage quickly because of the “don't eat-me” signals (Ravichandran, 2010). Under some circumstances, however, phagocytes kill live cells. For instance, macrophages induce apoptosis of normal vascular endothelial cells of the hyaloid vascular system as well as papillary cells in the developing mouse eye (Lang and Bishop, 1993; Diez-Roux and Lang, 1997). Similarly, microglial phagocytosis has been reported to induce the death of viable, motile non-apoptotic polymorphonuclear neutrophils in organotypic hippocampal cultures in which ischemia was induced by oxygen and glucose deprivation (Neumann et al., 2008). Further, in inflammatory conditions driven by activation of TLR2 or TLR4, microglial phagocytosis induces cerebellar granule cell death by a complex mechanism involving the microglial release of peroxynitrite which leads to a transient exposure of PS in the neurons and opsonization with MFG-E8, followed by recognition through the vitronectin receptor and phagocytosis by microglia *in vitro* (Neher et al., 2011) and in the striatum *in vivo* (Fricker et al., 2012). However, high concentrations of peroxynitrite lead to increased intracellular calcium, exposure of PS, activation of caspases and, ultimately, apoptotic cell death (Leist et al., 1997). Neurons dying from primary phagocytosis do not express features of apoptosis or necrosis, and the actual mechanism executing death is an open area of research (Brown and Neher, 2012). Thus, phagocytosis represents a wide range of responses, from the mere passive clearing of apoptotic cells and the active execution of final stages of apoptosis during development to the aberrant killing of live, healthy neurons during inflammation.

## Microglial phagocytosis in pathological conditions

**Figure 4:**
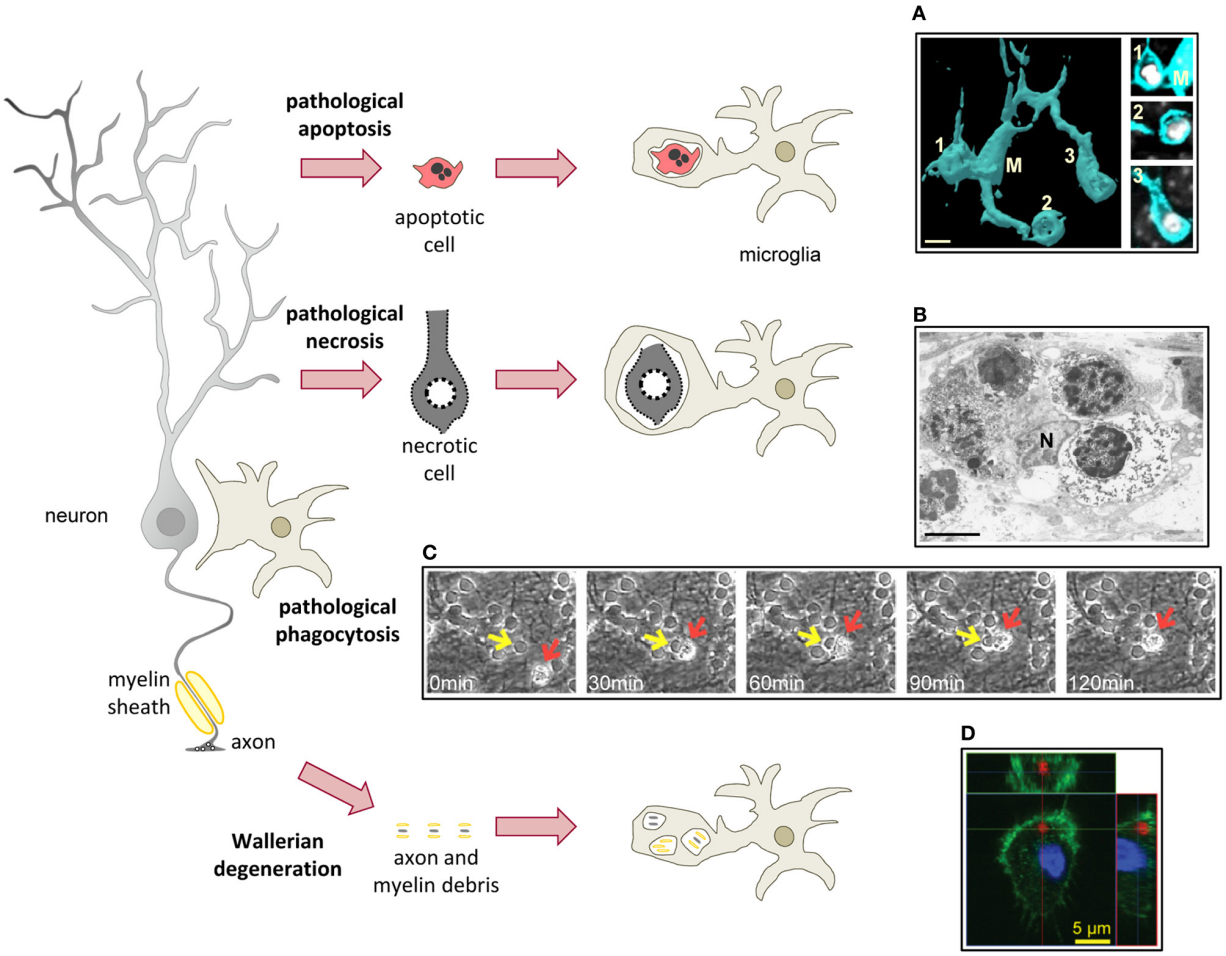
**Microglial phagocytosis in disease.** In pathological conditions microglia is challenged and usually assumes a hypertrophic morphology. Challenged microglia phagocytose apoptotic and necrotic cells **(A,B)**. In some inflammatory conditions, microglial phagocytosis can kill healthy neurons **(C)**. Microglia also phagocytose axonal and myelin debris resulting from Wallerian degeneration of severed axons **(D)**. **(A)** Surface-rendered fms-EGFP microglia (M; cyan) from the adult hippocampus phagocytosing three apoptotic/pycnotic cells (labeled with DAPI, white), in a mouse systemically challenged with bacterial lipopolysaccharides (5 mg/kg). **(B)** Electron microscopy microphotograph of embryonic (E20) microglia (N, microglial nucleus) from the rat cingulate cortex following maternal hypoxia (1 day) containing four engulfed dead cells. In the developing brain, hypoxia induces a whole range of death mechanisms, including apoptosis, pathological apoptosis, and necrosis (Blomgren et al., 2007). **(C)** Time-lapse imaging of microglia (red arrow) engulfing a live neuron (yellow arrow) in a co-culture challenged with the TLR2 agonist lipoteichoich acid (50 μg/ml). **(D)** Orthogonal projection of a confocal z-stack showing the engulfment of degenerated axons (labeled with tdTomato, red) by microglia (labeled with Iba1, green; nucleus labeled with DAPI, blue) in a co-culture model. Scale bars, 5 μm. Image **(A)** is reprinted with permission from Elsevier from Sierra et al. (2010). Image **(B)** is reprinted with permission from Elsevier from Li et al. (1998). Image **(C)** is reprinted with permission from Neher et al. (2011), Copyright 2011. The American Association of Immunologists., Inc. Image **(D)** is reprinted with permission from Hosmane et al. (2012).

Overall, phagocytosis is considered a beneficial phenomenon and its alteration has been linked to autoimmune diseases (Nagata et al., 2010). In addition, the best-known case of a phagocytic system disease is the relatively rare but lethal Nasu–Hakola disease or polycystic lipomembranous osteodysplasia with sclerosing leukoencephalopathy (PLOSL), due to loss-off-function mutations of TREM2 and/or DAP12 (Paloneva et al., 2002). PLOSL is characterized by defects in bone resorption by osteoclasts leading to the formation of cysts, as well as dementia (Bianchin et al., 2004). The contribution of microglia to the pathology of PLOSL remains obscure because DAP12-deficient mice do not reflect the human neurodegenerative pathology. Initial reports showed altered synaptogenesis and hypomyelination as well as behavioral impairments in mice deficient or expressing loss-off-function mutations of DAP12 (Kaifu et al., 2003; Roumier et al., 2004). In the mouse brain, DAP12 is exclusively expressed in microglia, suggesting an interesting link between microglia and synaptic plasticity through a yet unknown mechanism (Roumier et al., 2004). More recent studies have shown a strong demyelination in the brain of DAP12-deficient human patients; however this demyelination did not seem to correlate with changes in microglial density or activation (Satoh et al., 2011). Therefore, the exact pathophysiology of microglia in PLOSL patients remains unclear. Nonetheless, there are many other pathological conditions related to the microglial phagocytosis of apoptotic cells, viral and bacterial pathogens, tumor cells, Aβ, myelin, and axonal debris (Figure 4).

### Microglial phagocytosis of dead cells in pathological conditions

Neurodegeneration by apoptosis is a major part of several brain diseases such as stroke, epilepsy, prion disease, Alzheimer's disease (AD), and Parkinson's disease (PD). Microglial phagocytosis of apoptotic cells in pathological conditions is assumed to be highly efficient, possibly because of an extrapolation of the physiological phagocytosis during brain development, however this may not be always the case. For instance, there is qualitative evidence that apoptotic neurons in a mouse model of neonatal stroke by medial cerebral artery occlusion (MCAO) are poorly phagocytosed by activated microglia compared to the contralateral healthy hemisphere, where less apoptosis occurs (Faustino et al., 2011). The reasons behind this low phagocytosis in stroke are unknown, but it has been speculated that high levels of pro-inflammatory cytokines produced by astrocytes and vessels may be the culprit (Faustino et al., 2011). However, microglial phagocytosis of apoptotic cells *in vitro* is increased by the pro-inflammatory interferon gamma (IFNγ), but it remains unaltered by TNFβ or TGFβ (Chan et al., 2001). Prion disease is another condition where microglial phagocytosis is impaired. *In vitro*, the pathogenic form of prion protein (PrP^Sc^), reduces the phagocytosis of latex beads (Ciesielski-Treska et al., 2004); however *in vivo* (ME7 model of prion disease) microglia show increased phagocytosis of beads, and enhanced expression of the phagocytic machinery genes, including scavenger receptors, cathepsins, and proteins of the respiratory burst. In addition, qualitative phagocytosis of neuronal debris by microglia suggests that the microglial phagocytic machinery in prion disease is not disrupted (Hughes et al., 2010), but microglia do fail to phagocytose prion protein (PrP^Sc^), which accumulates in the brain and leads to neurodegeneration (Hughes et al., 2010). PD is one more example in which the phagocytosis behavior of microglia is altered. Here the phagocytosis of latex beads is prevented by the aggregated form, and enhanced by the monomeric form of alpha synuclein, which are found in the parenchyma and the CSF of parkinsonian patients, respectively (Park et al., 2008). Moreover, peripheral blood monocytes from PD patients, compared to age-matched controls, exhibit decreased phagocytosis of latex beads *ex vivo* (Salman et al., 1999), however defects in microglial phagocytosis have not been reported *in vivo* so far. Again, the lack of a standardized approach to quantify microglial phagocytosis hampers our understanding of its role in brain disorders where it is expected to play a significant role in removing cellular debris.

Death by necrosis is also an important component of many brain diseases. Contrary to apoptosis, necrosis is characterized by bursting of the cell membrane and spillover of cellular contents (Savill et al., 2002). Necrosis remains an obscure process, and molecular details of its execution are not well-known. Two main forms have been recognized: accidental necrosis, or cell lysis, by exposure of toxins, physical damage, freezing, etc.; and necrosis-like programmed cell death (PCD), which involves specialized caspase-independent signaling pathways such as the apoptosis-inducing factor (AIF) or cathepsins, and evolves without chromatin condensation (Leist and Jaattela, 2001). While accidental necrosis is unavoidable unless the stimulus is removed, cells can be rescued from necrotic PCD. The mechanisms of necrotic cells recognition by macrophages and microglia are not fully understood but may involve similar signaling pathways to the recognition of apoptotic cells. For instance, in some forms of necrotic PCD, exposure of PS by calcium-independent signals triggers recognition of necrotic cells by macrophages and microglia (Hirt et al., 2000; Hirt and Leist, 2003). One alternative mechanism could be the passive release of high mobility group 1 protein (HMGB1) by necrotic cells, which binds to the receptor for advanced glycation end products (RAGE) in phagocytes and triggers inflammation (Scaffidi et al., 2002). It is worth noting that HMGB1 is not released by apoptotic cells even when they fail to be phagocytosed and transform into secondary necrotic cells (Scaffidi et al., 2002). HMGB1 is also released during ischemia (assumedly, from the necrotic core) in stroke patients and in mice subjected to MCAO, launched an inflammatory response, which is detrimental for neuronal survival (Kim et al., 2006; Muhammad et al., 2008). Initial reports suggested that microglia does not mount an inflammatory response when co-cultured with necrotic PC12 neurons (De Simone et al., 2003). Others have shown that the phagocytosis of necrotic PCD Jurkat cells reduces the release of TNFα by microglia (Hirt and Leist, 2003). More recently, it has been shown that when co-cultured with necrotic HT22 neurons microglia do indeed express higher levels of pro-inflammatory cytokines (TNFα, IL-6), pro-inflammatory enzymes (nitric oxide synthase, 2-cyclooxygenase), MHC-II, and the integrin CD11b through a mechanism involving MYD88, a TLR adapter protein (Pais et al., 2008). In summary, very little is known about the efficiency and consequences of microglial phagocytosis of necrotic cells.

Death of living cells by phagocytosis, recently termed “phagoptosis,” might contribute to the pathophysiology of some diseases where inflammation, the main trigger for phagoptosis, occurs. For instance AD, which characterized at the histological level by plaques of Aβ as well as neurofibrillary tangles, has a strong inflammatory component (Johnston et al., 2011). *In vitro*, low concentrations of Aβ (1–42) (nanomolar range) lead to activation of microglia and an inflammatory response, which is partly responsible for neuronal damage (Maezawa et al., 2011). In this situation, either depleting microglia, blocking vitronectin receptors, inhibiting cytoskeleton polymerization, or preventing recognition of PS with annexin V, all prevent neuronal death induced by nanomolar Aβ (Neniskyte et al., 2011). These data suggest that phagoptosis might be partly responsible for neuronal death induced by Aβ, perhaps providing an explanation for the low numbers of dead neurons found in AD (Neniskyte et al., 2011). Nonetheless, death by apoptosis has been documented in AD patients and in animal models of AD, although it is assumed to occur at low levels over a long period of time (Shimohama, 2000). We argue that the clearance time for phagoptotic neurons is likely to be longer than for phagocytosed apoptotic neurons, because phagoptotic cells fail to activate caspases and other mechanisms of self-destruction and rely exclusively on microglia for degradation (Brown and Neher, 2012). Time-lapse imaging experiments suggest that engulfing a live neuron may take microglia under 2 h (Neher et al., 2011) (Figure 4), however the time to fully degrade it has not been estimated. As a longer clearance time implies a higher probability of phagocytosis detection, it should then be possible to visualize and quantify microglial engulfment of live cells in AD or in other inflammatory diseases to ultimately determine the contribution of phagoptosis to brain pathologies *in vivo*.

### Microglial phagocytosis of invading microorganisms

In the adult and developing brain, microglia are capable of phagocytosing many pathogens, including bacteria (*Escherichia coli, Streptococcus pneumonia, Staphylococcus aureus, Enterococcus faecalis*), yeast (*Saccharomyces cerevisiae*), and fungus (*Candida albicans*) (Kaur et al., 2004; Falsig et al., 2008; Shah et al., 2008; Hadas et al., 2010; Ribes et al., 2010; Peppoloni et al., 2011; Kochan et al., 2012). Different PAMPs are recognized by different receptors: for instance, recognition of *S. aureus* peptidoglycan is mediated by TLR2 (Kielian et al., 2005); recognition of *E. coli* LPS is mediated by TLR4 (Sivagnanam et al., 2010); and recognition of *S. cerevisiae* β-glucans (such as zymosan) is mediated by Dectin1, CR3, and the mannose receptor (MR, CD206) (Hadas et al., 2010). TLR2 and 4 are constitutively expressed by microglia (Bsibsi et al., 2002) and recognition of their ligands enhances phagocytosis (Ribes et al., 2010; Kochan et al., 2012), supporting the capacity of microglia to efficiently engulf and degrade infecting microorganisms. For instance, *E. coli* bacteria injected into the corpus callosum of early postnatal rats are engulfed in large numbers 1–3 h after injection, and are completely eliminated in 24 h (Kaur et al., 2004). In contrast, phagosomes were found within microglial cells up to 7 days after (Kaur et al., 2004), suggesting that degradation and killing of living microorganisms is a time-consuming process. Phagocytosis of bacteria activates the inflammatory cascade in microglia, inducing the expression of pro-inflammatory cytokines (TNFα), TLRs (TLR4, TLR9), complement receptors (CR3), scavenger receptors (SRA), and MHC-II (Sivagnanam et al., 2010). Although the data on microglial phagocytosis of microorganisms is not abundant, it seems to suggest a high capacity to fight against brain infections such as meningitis.

### Microglial phagocytosis of tumor cells

Brain tumors are very aggressive and have extremely poor prognosis possibly due to a failure of the innate and adaptive immune response to efficiently eliminate them. Tumor-associated microglia and macrophages (TAMs) are found in large numbers in human glioblastoma and have a complex and bidirectional relationship with glioma cells which has been reviewed in detail elsewhere (Watters et al., 2005). On one hand, glioma-initiating cells contribute to the recruitment of TAMs into the tumor mass (Yi et al., 2011) and polarize them toward a M2 phenotype (Wu et al., 2010). On the other hand, TAMs contribute to glioma progression by enhancing tumor migration and proliferation through growth factors, angiogenic molecules, and enzymes degrading the extracellular matrix (Watters et al., 2005). Unfortunately, glioma cells are rarely phagocytosed by TAMs in mouse models of glioma (Galarneau et al., 2007). Microglia from human glioblastoma patients do have the capability of phagocytosing latex microbeads *ex vivo* (Hussain et al., 2006), however *in vitro* studies have shown that co-culture with tumor cells decreases bead phagocytosis after a transient increase (Voisin et al., 2010). Further, it seems that tumor cells evade phagocytosis by lacking in their membrane the appropriate “eat-me” signals. In fact, microglia do phagocytose tumor cells which have been previously induced apoptosis with the cytotoxic agent etoposide (Chang et al., 2000) or UV light (Kulprathipanja and Kruse, 2004), although many tumor cells decrease their sensitivity to apoptosis inducers during oncogenic transformation (Maher et al., 2001). This data has suggested the use of alternative therapies to promote tumor cell apoptosis, such as oncolytic viruses, which selectively replicate in tumor cells, leading to their destruction (Zeyaullah et al., 2012). This therapy, while safe, has been proven ineffectual because TAMs clear the viruses away from the tumor (Fulci et al., 2007). While the literature confirms that TAM phagocytosis of tumor cells is not very effective, others have suggested that some tumor cells, particularly in highly invasive tumors such as glioblastoma, phagocytose neighboring cells perhaps as a way to fuel their constant growing [reviewed by Huysentruyt and Seyfried (2010)].

### Microglial phagocytosis of Aβ deposits

Aβ is a small peptide produced by proteolytic cleavage from amyloid precursor protein (APP) by β- and γ-secretases. The most pathogenic form is Aβ (1–42), which forms fibrils, insoluble aggregates found in amyloid plaques in the brains of AD patients, as well as human immunodeficiency virus (HIV)-associated neurocognitive disorders (Xu and Ikezu, 2009). A major effort has been put into developing therapies to lower the amyloid burden. Aβ reduction can occur either by decreasing its synthesis rate (e.g., with secretase inhibitors); or by increasing its elimination rate. The particular location of microglia surrounding plaques in human patients and mouse models of AD lead to the early suggestion that they could be responsible for releasing Aβ and forming the plaques (Lai and McLaurin, 2012). While microglia can synthesize Aβ *in vitro* (Banati et al., 1993), APP mRNA is not found in microglia of human AD brains (Scott et al., 1993). It was alternatively proposed that microglia could be responsible for phagocytosing Aβ and contribute to its clearance (Paresce et al., 1996). *In vitro*, microglia recognizes and engulfs fluorescently labeled fibrillary Aβ through a variety of receptors, including scavenger receptors (Paresce et al., 1996), TLR2 (Liu et al., 2012), and the TLR4-interacting molecule, CD14 (Liu et al., 2005). Further, Aβ induces a positive chemotaxis of microglia via TGFβ (Huang et al., 2010), possibly explaining their location around plaques. Recent live imaging experiments have shown that microglia is rapidly attracted to already formed plaques in a mouse model of AD with mutated APP and presenilin 1 (a protein of the γ-secretase complex) (Meyer-Luehmann et al., 2008), strongly suggesting that microglia does not participate in the initial stages of plaque formation and confirming the chemotactic nature of Aβ. Importantly, the size of the plaques remained constant and no evidence of phagocytosis or plaque clearance was obtained (Meyer-Luehmann et al., 2008). In agreement, a detailed 3D reconstruction of microglia and amyloid fibrils in APP mutated mice showed that microglial processes and amyloid fibrils were interlaced forming a network, but Aβ was not found within microglia, further suggesting that microglia does not phagocytose Aβ *in vivo* (Stalder et al., 2001). The failure of microglia to clear Aβ plaques of AD patients is unclear. Cultured microglia engulf and partially degrade Aβ *in vitro* over the first 3 days, but no additional degradation and a slow release of intact Aβ is found afterwards (Chung et al., 1999). Furthermore, microglia from old mutant APP/presenilin 1 mice have a decreased expression of phagocytic genes (SRA, CD36, RAGE, etc.) and increased expression of pro-inflammatory cytokines (TNFα, IL-1β), compared with age-matched wild type mice (Hickman et al., 2008), but both findings cannot explain the failure of microglia to sufficiently clear Aβ plaque *in vivo*. The role of inflammation in Aβ clearance is also not resolved. *In vitro*, inflammatory challenge by LPS, TNFα, IL-1β, or IFNγ inhibits fibrillary Aβ phagocytosis, whereas anti-inflammatory cytokines had no effect (Koenigsknecht-Talboo and Landreth, 2005). Aβ phagocytosis is blocked by the anti-inflammatory celecoxib (Persaud-Sawin et al., 2009), but not by minocyline (Familian et al., 2007). *In vivo*, LPS administered locally either transiently reduced (Herber et al., 2004), or enhanced (Qiao et al., 2001) Aβ load in mouse models of AD. The fractalkine receptor, a regulator of phagocytosis, has been found to contribute to the Aβ burden by some (Lee et al., 2010; Liu et al., 2010) but not others (Fuhrmann et al., 2010). Importantly, common treatments for HIV and AD may be detrimental for Aβ clearance, at least *in vitro*. HIV protease inhibitors either block Aβ degradation or enhance secretion of non-degraded Aβ by macrophages (Lan et al., 2012). Similarly, γ-secretase inhibitors prevent microglial Aβ engulfment (Farfara et al., 2011). Another point of controversy is the participation of invading macrophages. The blood-brain barrier (BBB) is partially disturbed in AD patients, facilitating the extravasation of circulating monocytes (Lai and McLaurin, 2012). Macrophages have higher Aβ capacity intake than microglia *in vitro* (Lai and McLaurin, 2012), and ablation experiments have suggested that the Aβ burden is cleared by blood-borne macrophages, but not resident microglia (Simard et al., 2006). The issue is further complicated by the fact that experimental interventions such as irradiation of mice lead to invasion of blood-monocytes into the brain parenchyma and that macrophages and microglia are phenotypically indistinguishable by surface markers.

### Microglial phagocytosis of myelin and axonal debris

The only available data on microglial phagocytosis of axonal debris comes from *in vitro* models. In cortical explants where growing neurites were sectioned, the debris was cleared by added microglia (Tanaka et al., 2009). In a compartmentalized co-culture model where neurons were grown in a chamber and their axons extended in bundles through microchannels, axotomy, or nitric oxide treatment-induced axonal degeneration and microglia rapidly cleared the axonal debris (Hosmane et al., 2012). Interestingly, the mechanisms of recognition of axonal debris seem to be different from those of apoptotic cells (Tanaka et al., 2009). A candidate receptor is TIR domain-containing adapter inducing interferon beta (TRIF). The clearance of axonal debris is prevented by blocking TRIF signaling *in vitro*, and TRIF-deficient mice have a smaller percentage of microglia containing neurofilament-positive axonal material after dorsal root axotomy (Hosmane et al., 2012). In both set ups, axonal debris (lacking myelin) had a detrimental effect on axon regrown, which was prevented by microglial phagocytosis (Tanaka et al., 2009; Hosmane et al., 2012), suggesting that enhancing microglial phagocytosis is a novel therapeutical tool in traumatic brain injuries.

More attention has been put into the mechanisms of myelin debris clearance, particularly during MS and in spinal cord and nerve injuries. In spinal cord injury, the degeneration of the severed axons through anterograde or Wallerian degeneration (WD) is followed by degradation of myelin and apoptosis of myelinating cells, oligodendrocytes (Crowe et al., 1997). The etiology of MS remains to be fully elucidated and several hypotheses have been proposed to explain the demyelination found in patients, from an autoimmune attack to myelin followed by axonal degeneration, to the developmental, environmental, or virus-induced degeneration of axons by WD followed by myelin degradation; in both cases, myelin debris accumulates (Stys et al., 2012). Myelin proteins such as Nogo are well-known to interfere with axonal regeneration and repair, therefore an efficient myelin clearance is an absolute requirement for recovery (Wang et al., 2002). Furthermore, myelin inhibits its own phagocytosis through myelin CD47 binding to microglia and macrophages signal regulatory protein α (SIRPα) (Gitik et al., 2011). Contrary to the peripheral nervous system (PNS), myelin clearance after WD is very inefficient in the CNS [reviewed in Gaudet et al. (2011)]. In PNS lesions, the myelinating Schwann cells are the major phagocytic population in the first few days, followed by invading macrophages (Hirata and Kawabuchi, 2002). Importantly, macrophages express Nogo receptors NgR1 and NgR2, which facilitate their migration out of the healing nerve, thus resulting in the resolution of the inflammatory response (Fry et al., 2007). In culture, myelin-phagocytosing macrophages inhibit T cell proliferation, further containing the immune response (Bogie et al., 2011). The rapid clearance of myelin and resolution of the immune response is greatly responsible for regeneration after nerve injury.

In contrast, CNS regeneration after trauma does not occur, and microglial poor phagocytosing capabilities together with poor or slow recruitment of macrophages are partly to blame (Gaudet et al., 2011). *In vitro*, microglia recognize and phagocytose myelin through CR3, SRA, and FcRs among other receptors (Smith, 2001). Microglia do phagocytose myelin to some extent in mouse models of MS, a phenomenon which is stimulated by the presence of MBP-reactive T cells (Nielsen et al., 2009). However, myelin debris is still observed in the human spinal cord years after the injury (Buss et al., 2004). Together with insufficient activation of microglia, the absence of autoreactive antibodies, and the subsequent lack of activation of FcRs in traumatic brain injury have been suggested to explain why microglia phagocytose myelin in MS but not in spinal cord injury (Rotshenker, 2003). Microglial phagocytosis of myelin after hemisection of the ascending sensory tract is increased after treating the mice with LPS, resulting in decreased myelin debris, but, unfortunately, no axonal regeneration (Vallieres et al., 2006), possibly because of the pro-inflammatory phenotype induced by LPS. More recently, a microglial phenotype supportive of remyelination has been described (Olah et al., 2012). During the remyelination phase after cuprizone-induced demyelination in the corpus callosum, a mouse model of MS, myelin-phagocytosing microglia express genes involved not only in phagocytosis but also in the activation, migration, proliferation, and differentiation of oligodendrocytes precursor cells (Olah et al., 2012). While it remains to be directly assessed whether myelin phagocytosis triggers this remyelination-supportive phenotype, this data suggests that the beneficial consequences of enhancing microglial phagocytosis of myelin may be two-fold: clearing myelin and facilitating remyelination.

## Conclusion

In conclusion, microglial phagocytosis is a pivotal mechanism of clearance of cellular debris in health and disease. Like Janus, the roman god of war and peace, microglial phagocytosis has beneficial (e.g., anti-inflammatory) and detrimental (e.g., respiratory burst) consequences for tissue homeostasis which remain largely unexplored *in vivo*. In particular, the establishment of standardized methods to quantify microglial phagocytosis of different types of cargo, as well as the development of novel tools to specifically block recognition, engulfment, and degradation of cargo, will undoubtedly delineate the ultimate impact of microglial phagocytosis *in vivo*.

### Conflict of interest statement

The authors declare that the research was conducted in the absence of any commercial or financial relationships that could be construed as a potential conflict of interest.

## Abbreviations

ACAMPs: apoptotic cells-associated cellular patterns
AD: Alzheimer's disease
AIF: apoptosis-inducing factor
APCs: antigen presenting cells
APP: amyloid precursor protein
ASD: autism spectrum disorders
ATP: adenosine trinucleotide phosphate
Aβ: amyloid beta
BAI-1: brain-specific angiogenesis inhibitor 1
CAD: caspase-activated DNAse
CNS: central nervous system
CR1: complement receptor 1
DAP12: DNAX-activation protein X
DCs: dendritic cells
DOCK-180: dedicator of cytokinesis 180
EAE: experimental acute encephalomyelitis
ELMO: engulfing and cell motility protein
HMGB1: high mobility group 1 protein
Hsp60: heat shock protein 60
IFNγ: interferon gamma
IGF-1: insulin-like growth factor 1
IL-10: interleukin 10
IL-1β: interleukin 1beta
IL-4: interleukin 4
LPS: bacterial lipopolysaccharides
MBP: myelin basic protein
MCAO: medial cerebral artery occlusion
MFG-E8: milk fat globule-epidermal growth factor
MHC-II: major histocompatibility complex class II
MS: multiple sclerosis
NADPH: nicotinamide adenine dinucleotide phosphate
NGF: neural growth factor
PAMPS: pathogen-associated molecular patterns
PCD: programmed cell death
PD: Parkinson's disease
PLOSL: polycystic lipomembranous osteodysplasia with sclerosing leukoencephalopathy
PNS: peripheral nervous system
PS: phosphatidylserine
RAGE: receptor for advanced glycation end products
ROS: reactive oxygen species
RPE: retinal pigment epithelium
SIRPα: signal regulatory protein alpha
SVZ: subventricular zone
TAMs: tumor-associated macrophages
TGFβ: transforming growth factor beta
TLRs: Toll-like receptors
TNFα: tumor necrosis factor alpha
TREM2: triggering receptor expressed on myeloid cells-2
TRIF: TIR domain-containing adapter inducing interferon beta
UDP: urydil dinucleotide phosphate
UTP: urydil trinucleotide phosphate
WD: Wallerian degeneration.
